# Supplementary material for: Familial Risks for Liver, Gallbladder and Bile Duct Cancers and for Their Risk Factors in Sweden, a Low-Incidence Country
Source: Cancers (Basel). 2022 Apr 12;14(8):1938. doi: 10.3390/cancers14081938 (PMC9030935; doi:10.3390/cancers14081938)
Supplement: Supplementary file 1 [file cancers-14-01938-s001.zip › cancers-1607942-supplementary.pdf]

Supplementary materials

Table S1. ICD codes of comorbidities and patient numbers, 1964-2018**.**

	**ICD-7 (1964-1968)**	**ICD-8 (1969-1986)**	**ICD-9 (1987-1996)**	**ICD-10 (1997-)**	**No.**	**%**
Diabetes	260	250	250	E10-E14	759476	26.0
Autoimmune hepatitis				K75.4	1888	0.1
Primary biliary cirrhosis	-	-	571G	K74.3	3415	0.1
Hepatitis B virus	-	070.2, 070.3	070C-D	B18.0, B18.1	13543	0.5
Hepatitis C virus	-			B17.1, B18.2	22350	0.8
Gallstone disease	584, 585	574, 575	574, 575	K80, K81	473657	16.2
Obesity	287	277.99	278A	E65-E68	201353	6.9
Alcohol related disease	307, 322, 581 (ex 581.0)	291, 303, 571 (ex 571.8)	291, 303, 571A-D	F10, K70	418604	14.3
Non-alcohol related disease	581.0	571.8	571F, 571X	K75.8, K76.0	6922	0.2
Other hepatitis	092, 580	570	570, 571E	B17.8, B17.9, B18.8, B18.9, B94.2, K73.0-9, K75.9	11442	0.4
Infection of bile ducts.	586	576	576	K83	51739	1.8
Chronic obstructive pulmonary disease	500-502	490-493	490-496	J40-J47	961024	32.9
All of above					2925413	100.0

Table S2. Study population and number of case of hepatobiliary cancer with and without family history of hepatobiliary cancer, 1958-2018

	With family history of hepatobiliary cancer		Non family history of hepatobiliary cancer	
	No.	%	No.	%
**Population (N=9338882)**	94208	100.0	9244674	100.0
Men (N=4792416)	48158	51.1	4744258	51.3
Women (n=4546466)	46050	48.9	4500416	48.7
**Total case of hepatobiliary cancer**				
N. of case (N=8862) (% of all cases)	304	3.4	8558	96.6
Mean age at diagnosis (± SD)	63.9 ± 10.2		61.7 ± 13.9	
Incidence rate (per 100 000 person years)*, 95% CI	13.0, 11.6-14.5		9.4, 9.2-9.6	
**Men**				
N. of case (N=5127)	168	55.3	4959	57.9
Mean age at diagnosis (± SD)	63.6 ± 10.3		61.4 ± 13.8	
Incidence rate (per 100 000 person years)*, 95% CI	14.3, 12.1-16.5		10.8, 10.5-11.1	
**Women**				
N. of case (N=3735)	136	44.7	3599	42.1
Mean age at diagnosis (± SD)	64.4 ± 10.2		62.1 ± 14.1	
Incidence rate (per 100 000 person years)*, 95% CI	11.8, 9.8-13.8		8.1, 7.8-8.3	
**Subtypes of hepatobiliary cancer (N=8862)**	304	100.0	8558	100.0
HCC (N=5315)	189	62.2	5126	59.9
Gall bladder (N=1878)	76	25.0	1802	21.1
Extrahepatic bile ducts (N=1172)	29	9.5	1143	13.4
Ampulla of Vater (N=497)	10	3.3	487	5.7

*: Adjusted for European standardized population;
CI: Confidence interval; ICD: International classification of diseases.

Table S3. Familial risks of concordant hepatobiliary cancer in men and women

Hepatobiliary cancer in family	HCC				Gall bladder				Extrahepatic bile ducts				Ampulla of vater			
	O	SIR	95% CI		O	SIR	95% CI		O	SIR	95% CI		O	SIR	95% CI	
**Men**																
HCC	88	**2.73**	**2.19**	**3.36**	7	1.65	0.65	3.41	4	0.68	0.18	1.76	3	1.24	0.23	3.68
Gall bladder	30	1.14	0.77	1.63	10	**2.75**	**1.31**	**5.07**	6	1.19	0.43	2.60	2	0.96	0.09	3.55
Extrahepatic bile ducts	7	0.82	0.33	1.71	2	1.73	0.16	6.36	2	1.26	0.12	4.62	2	3.07	0.29	11.27
Ampulla of Vater	5	1.37	0.43	3.21	0				0				0			
All	130	**1.84**	**1.54**	**2.18**	19	**1.99**	**1.20**	**3.12**	12	0.91	0.47	1.60	7	1.29	0.51	2.68
**Women**																
HCC	34	**2.32**	**1.60**	**3.24**	17	1.36	0.79	2.18	6	1.21	0.44	2.65	3	1.56	0.29	4.62
Gall bladder	20	**1.73**	**1.06**	**2.68**	29	**2.76**	**1.85**	**3.97**	6	1.44	0.52	3.16	0			
Extrahepatic bile ducts	3	0.79	0.15	2.33	11	**3.23**	**1.60**	**5.80**	4	2.97	0.77	7.68	0			
Ampulla of Vater	2	1.25	0.12	4.58	0				1	1.81	0.00	10.36	0			
All	59	**1.86**	**1.42**	**2.41**	57	**2.05**	**1.55**	**2.66**	17	1.54	0.90	2.48	3	0.71	0.13	2.10

O=Observed; SIR=Standardized incidence ratio; CI=Confidence intervals.
Bold types: 95% CI does not include 1.00.

Table S4. Familial risks of hepatobiliary cancer of family members with any cancer in men and women

	HCC				Gall bladder				Extrahepatic bile ducts				Ampulla of Vater			
Cancer in family	O	SIR	95% CI		O	SIR	95% CI		O	SIR	95% CI		O	SIR	95% CI	
**Men**																
Pancreas	81	1.12	0.89	1.39	13	1.33	0.71	2.28	30	**2.20**	**1.48**	**3.15**	9	1.61	0.73	3.07
Colon	205	0.94	0.82	1.08	31	1.08	0.73	1.53	51	1.26	0.94	1.66	27	**1.61**	**1.06**	**2.35**
Lung	268	**1.38**	**1.22**	**1.55**	37	**1.48**	**1.04**	**2.04**	31	0.89	0.60	1.26	20	1.40	0.85	2.16
Hepatobiliary	130	**1.84**	**1.54**	**2.18**	19	**1.99**	**1.20**	**3.12**	12	0.91	0.47	1.60	7	1.29	0.51	2.68
Breast	287	0.95	0.84	1.07	49	1.24	0.91	1.63	50	0.91	0.68	1.20	33	**1.45**	**1.00**	**2.04**
Cervix	43	1.12	0.81	1.51	5	1.03	0.32	2.41	4	0.59	0.15	1.52	4	1.44	0.37	3.72
Melanoma	59	1.01	0.77	1.31	8	1.07	0.46	2.12	19	**1.85**	**1.11**	**2.90**	4	0.95	0.25	2.45
Nervous system	42	0.94	0.68	1.27	6	1.03	0.37	2.26	9	1.12	0.51	2.14	8	**2.45**	**1.05**	**4.85**
All	2344	**1.04**	**1.00**	**1.08**	369	**1.24**	**1.12**	**1.37**	407	0.98	0.89	1.09	196	**1.15**	**1.00**	**1.32**
**Women**																
Pancreas	39	1.20	0.85	1.64	33	1.17	0.80	1.64	11	0.98	0.49	1.76	9	2.05	0.93	3.90
Colon	95	0.98	0.79	1.20	72	0.86	0.67	1.09	43	1.30	0.94	1.75	21	1.59	0.98	2.44
Lung	116	**1.33**	**1.10**	**1.60**	65	0.89	0.69	1.14	36	1.26	0.88	1.74	9	0.78	0.36	1.49
Hepatobiliary	59	**1.86**	**1.42**	**2.41**	57	**2.05**	**1.55**	**2.66**	17	1.54	0.90	2.48	3	0.71	0.13	2.10
Breast	108	0.80	0.65	0.96	109	0.97	0.80	1.17	47	1.06	0.78	1.41	14	0.76	0.42	1.29
Cervix	28	**1.61**	**1.07**	**2.33**	13	0.91	0.48	1.56	3	0.54	0.10	1.59	3	1.35	0.26	4.01
Melanoma	25	0.93	0.60	1.37	25	1.20	0.78	1.78	9	1.10	0.50	2.09	4	1.11	0.29	2.88
Nervous system	24	1.20	0.77	1.79	18	1.12	0.66	1.78	9	1.42	0.64	2.71	5	1.90	0.60	4.46
All	1033	1.02	0.96	1.09	875	1.03	0.96	1.10	346	1.03	0.92	1.14	134	0.99	0.83	1.17

O=Observed; SIR=Standardized incidence ratio; CI=Confidence intervals.

Bold types: 95% CI does not include 1.00.
